# Efficacy and Mechanisms Underlying MRI-guided High-definition Transcranial Direct Current Stimulation Combined With Computerized Cognitive Remediation Therapy for Improving Cognitive Impairments in Schizophrenia: Study Protocol for a Randomized Controlled Trial

**DOI:** 10.31083/AP46768

**Published:** 2026-02-02

**Authors:** Yange Wei, Shanyuan He, Peng Luo, Rongxun Liu, Hanshuo Su, Zengyuan Shen, Shuqi Feng, Yanran Wu, Guangjun Ji, Wei Zheng, Fei Wang, Chuansheng Wang

**Affiliations:** ^1^Department of Early Intervention, Mental Health and Artificial Intelligence Research Center, The Second Affiliated Hospital of Xinxiang Medical University, Henan Mental Hospital, 453002 Xinxiang, Henan, China; ^2^Peking University Sixth Hospital, Peking University Institute of Mental Health, NHC Key Laboratory of Mental Health (Peking University), National Clinical Research Center for Mental Disorders (Peking University Sixth Hospital), 100191 Beijing, China; ^3^School of Public Health, Henan Medical University, 453003 Xinxiang, Henan, China; ^4^Department of Psychiatry, The Affiliated Brain Hospital of Guangzhou Medical University, 510631 Guangzhou, Guangdong, China; ^5^Unit of Early Intervention, The Affiliated Brain Hospital of Nanjing Medical University, 210029 Nanjing, Jiangsu, China; ^6^Department of Psychiatry, Yale School of Medicine, New Haven, CT 06511, USA

**Keywords:** schizophrenia, transcranial direct current stimulation, cognitive remediation, cognitive impairments, clinical protocol

## Abstract

**Background:**

Schizophrenia primarily depends on pharmacotherapy, which has demonstrated limited efficacy in enhancing cognitive impairments. High-definition transcranial direct current stimulation (HD-tDCS) and computerized cognitive remediation therapy (CCRT) hold potential for improving cognitive impairments. This study aims to investigate the effects of combining HD-tDCS with CCRT on cognition and to explore the mechanisms of this approach in schizophrenia.

**Study Design:**

This is the protocol of a randomized controlled trial.

**Methods:**

Schizophrenia patients will be randomly assigned to one of 4 groups: HD-tDCS + CCRT group (Group 1), HD-tDCS group (Group 2), CCRT group (Group 3), and a control group (Group 4). The central electrode will be personalized using magnetic resonance imaging (MRI)-guided localization in the medial prefrontal cortex (mPFC). CCRT includes 6 therapeutic modules and 10 distinct tasks. Both HD-tDCS and CCRT will be administered once daily, 5 days per week, for 4 consecutive weeks, culminating in a total of 20 sessions. Assessments will occur at baseline (T0), after 10 sessions (T1), after 20 sessions (T2), and after 6 months of follow-up (T3). The primary outcome measure is the change in cognition. We will employ multimodal MRI, serum concentrations of brain-derived neurotrophic factor (BDNF) and glial cell line-derived neurotrophic factor (GDNF) to explore the underlying mechanisms.

**Expected Results:**

An involvement of mPFC and synaptic plasticity in response to HD-tDCS and CCRT is hypothesized.

**Conclusion:**

The study will provide empirical evidence for the effectiveness of combined therapy at an individual level, explore its mechanisms, and may ultimately result in personalized medicine.

**Clinical Trial Registration:**

ChiCTR2500102731, https://www.chictr.org.cn/hvshowprojectEN.html?id=276964&v=1.0.

## Main Points

1. High-definition transcranial direct current stimulation (HD-tDCS) targeting the medial prefrontal cortex (mPFC) holds promise for enhancing cognitive functions in patients with schizophrenia.

2. Combining HD-tDCS with computerized cognitive remediation therapy (CCRT) might lead to more significant cognitive enhancements than using each treatment separately.

3. The biological mechanisms underlying the synergistic effects of HD-tDCS and CCRT on cognitive functions will be explored.

## 1. Introduction

Schizophrenia is a severe, chronic, and disabling mental disorder with an etiology that remains largely elusive. Clinically, it is characterized by cognitive impairments, negative symptoms, and positive symptoms [1]. Cognitive impairment is recognized as a core feature of schizophrenia, affecting various domains including working memory, language, verbal fluency, attention, reading ability, processing speed, and non-verbal reasoning [2, 3]. These impairments significantly impact patients’ social functioning and daily living skills, further perpetuating the stigma associated with mental illness and creating a detrimental cycle [4]. Schizophrenia poses multifaceted societal burdens, primarily manifesting as economic challenges, caregiving demands, emotional stress, and enduring pressures [5, 6]. While pharmacological treatments for schizophrenia, such as antipsychotic medications, are well-established and primarily effective in alleviating positive symptoms, their efficacy in improving cognitive impairments remains limited [7, 8]. Certain antipsychotic medications, notably first-generation agents and clozapine, have been associated with detrimental cognitive effects, a consideration that is critical in clinical decision-making [9]. Furthermore, pharmacological treatments are often accompanied by substantial economic burdens for families, low adherence rates, and potential adverse effects, including extrapyramidal symptoms as well as endocrine and metabolic disturbances [10]. Consequently, it is imperative to explore non-pharmacological interventions, such as neuro-modulation therapy and cognitive remediation, to ameliorate cognitive impairments in individuals with schizophrenia.

Transcranial direct current stimulation (tDCS) represents a non-invasive neuromodulatory approach employed in the treatment of schizophrenia, characterized by its low cost and reliable safety profile [11, 12]. Its mechanism may involve modulating the resting state of neuron membranes, thus affecting neuronal excitability [13]. High-definition transcranial direct current stimulation (HD-tDCS) is an advanced tDCS technology, offering the distinct advantage of delivering electrical currents with greater precision to targeted brain regions, thereby enhancing both accuracy and durability of effects [14, 15]. Recent studies have demonstrated that tDCS can improve attention, working memory, and social cognitive abilities in individuals with schizophrenia [16]. HD-tDCS has been shown to assist healthy individuals in more accurately assessing their memory status and enhancing working memory [17, 18]. Furthermore, HD-tDCS has demonstrated potential in ameliorating cognitive deficits in individuals with chronic schizophrenia [19]. In contemporary research involving patients with schizophrenia, the anodal stimulation site for most tDCS/HD-tDCS interventions is typically the left dorsolateral prefrontal cortex. However, studies have observed that alterations in neural activity within the medial prefrontal cortex (mPFC) are associated with decreased accuracy in reality monitoring tasks among schizophrenia patients [20]. In this population, inadequate deactivation of the mPFC during working memory tasks may significantly contribute to memory impairments [21]. Consequently, HD-tDCS targeting mPFC holds promise for enhancing cognitive performance in patients with schizophrenia. However, it is important to acknowledge that the effects of non-invasive brain stimulation can be inconsistent, and the cognitive improvements it offers may be challenging to sustain over the long term [22, 23]. Therefore, investigating the integration of non-invasive brain stimulation with other cognitive interventions that have more enduring effects, such as cognitive remediation therapy (CRT), to achieve synergistic and sustained enhancements, represents a promising avenue for future research.

CRT is an evidence-based intervention that has been demonstrated to enhance cognition in schizophrenia [24]. This has facilitated the development of a computerized variant, known as computerized CRT, which seeks to augment patients’ cognitive abilities through digital training programs [25, 26]. Computerized cognitive remediation therapy (CCRT) utilizes computer technology to deliver both standardized and individualized training tasks, thereby allowing for more precise and tailored therapeutic interventions targeting the cognitive functions. Several pieces of evidence suggest that CCRT can enhance both cognitive abilities and social functioning in individuals with schizophrenia [27, 28]. Moreover, the cognitive and social improvements facilitated by CCRT have been observed to persist for up to 6 months post-therapy [29]. Despite these promising outcomes, the efficacy of CCRT varies significantly among patients [30]. Some research indicates that CCRT does not produce substantial cognitive improvements in certain cases of schizophrenia [31]. Consequently, further clinical and mechanistic investigations are warranted to elucidate the effectiveness of CCRT in schizophrenia.

Combining HD-tDCS with CCRT might lead to more significant cognitive enhancements than using each treatment separately. A preliminary study indicated that the combination of tDCS and CRT led to improvements in cognitive domains such as visual memory, processing speed, and working memory, with these enhancements persisting one month post-therapy [32]. Another study involving 49 participants demonstrated that CRT paired with active tDCS significantly improved working memory in patients with schizophrenia, compared with CRT with sham tDCS, with these improvements sustained over an extended period of 56 days [33]. However, concerning clinical symptomatology, one study reported that CRT combined with active tDCS did not yield significant therapeutic effects on clinical symptoms in schizophrenia patients when compared to CRT with sham tDCS [34]. It is worth noting that the aforementioned study had a limited sample size, potentially impacting its statistical power. Consequently, the researchers recommended conducting larger-scale controlled trials to more comprehensively evaluate the potential benefits of combining CRT with CCRT. Research on tDCS in conjunction with CRT has demonstrated a positive impact on cognitive enhancement in individuals with schizophrenia. Currently, there is a lack of direct research examining the combined use of HD-tDCS and CCRT in schizophrenia patients.

Previous research has demonstrated that both HD-tDCS and CCRT independently enhance cognitive function in individuals with schizophrenia. However, studies investigating their combined application remain scarce. Schizophrenia is widely recognized as a disorder characterized by dysconnectivity within brain networks, particularly involving functional abnormalities in key brain regions such as the limbic system, temporal lobe, and parietal lobe, which correlate with the severity of cognitive deficits [35, 36]. Functional magnetic resonance imaging (fMRI) is utilized to indirectly assess neural activity in patients with schizophrenia by measuring blood oxygen level-dependent signals, thereby facilitating a comprehensive examination of treatment-induced changes in brain activity [37]. Importantly, prefrontal-temporal tDCS has been shown to modulate dysfunctional network connectivity in patients with schizophrenia, underscoring the potential of neuromodulation to target circuit-level deficits [38]. Moreover, tDCS not only modulates the activity and connectivity of cortical and subcortical brain networks but also influences blood concentrations of biomarkers such as brain-derived neurotrophic factor (BDNF) and glial cell line-derived neurotrophic factor (GDNF). These systemic alterations collectively form the biological basis for its therapeutic effects [39]. BDNF and GDNF function as critical biomarkers for synaptic plasticity, a process involved in the pathogenesis and treatment of schizophrenia [40]. The hypothesized mechanisms underlying HD-tDCS, particularly its enhancement of long-term memory, include the upregulation of BDNF expression, activation of its signaling pathway, and improved synaptic plasticity [41]. Similarly, studies on CCRT have demonstrated its effectiveness in enhancing cognitive function in schizophrenia, accompanied by observed increases in serum GDNF levels [42]. Moreover, the literature also proposes that serum BDNF and GDNF levels might represent the central nervous system’s BDNF and GDNF expression profile [43, 44]. BDNF is essential for neuronal synthesis, differentiation, maintenance, and survival. Studies have demonstrated a correlation between decreased peripheral blood BDNF levels and cognitive impairments, such as attention deficits, in individuals with schizophrenia [45, 46, 47]. GDNF levels have been associated with working memory performance and attention deficits [48]. As far as we know, no research has investigated the combined effects of magnetic resonance imaging (MRI)-guided HD-tDCS targeting mPFC and CCRT on cognitive deficits, nor the biological mechanisms underlying their combined use in treating schizophrenia.

The current study aims to achieve three primary objectives. The primary aim of this study is to evaluate the synergistic effects of HD-tDCS and CCRT on cognitive abilities and clinical symptoms through a randomized controlled trial. We hypothesize that the combined HD-tDCS and CCRT intervention will result in greater cognitive improvement in individuals with schizophrenia compared to either intervention alone. The secondary aim is to evaluate the long-term impacts of HD-tDCS and CCRT after a six-month period. The third aim is to investigate the biological mechanisms of MRI-guided HD-tDCS and CCRT in schizophrenia, utilizing MRI, and measuring serum BDNF and GDNF levels. This study not only promises to contribute to the development of innovative therapeutic strategies for schizophrenia but also seeks to enhance our understanding of its pathophysiology.

## 2. Methods

### 2.1 Study Design 

**Fig. 1.**
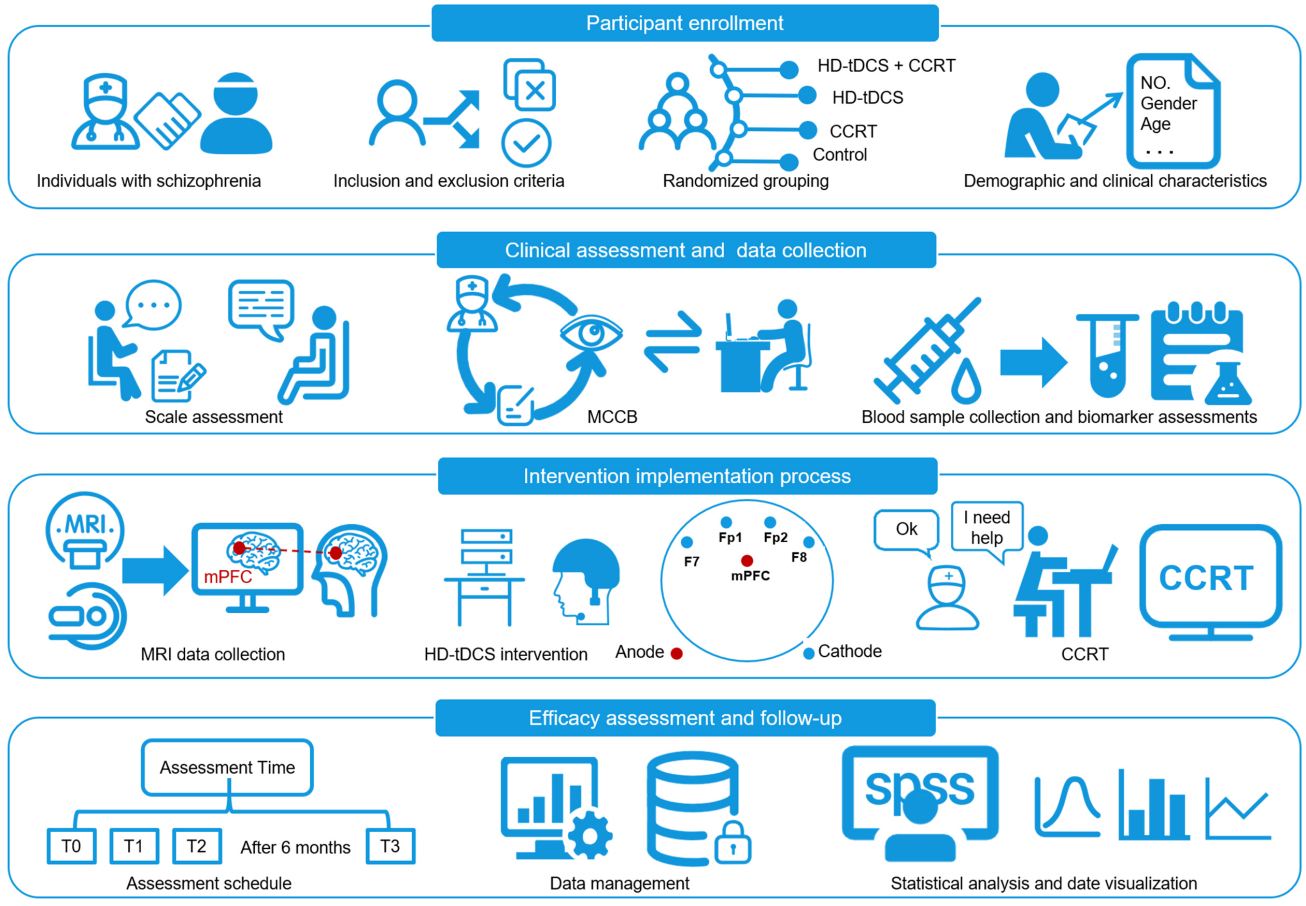
**Schematic overview of the study procedure**. CCRT, computerized cognitive remediation therapy; HD-tDCS, high-definition transcranial direct current stimulation; MCCB, MATRICS Consensus Cognitive Battery; MRI, magnetic resonance imaging. T0, baseline; T1, after 10 sessions; T2, after 20 sessions; T3, after 6 months of follow-up; mPFC, medial prefrontal cortex. The labels Fp1, Fp2, F7, and F8 denote standard electrode positions according to the international 10-20 system, indicating the placement of the cathode electrodes for the HD-tDCS montage.

This study is a prospective, double-blind, randomized controlled trial protocol to assess the efficacy of HD-tDCS combined with CCRT in enhancing cognitive function among patients with schizophrenia. Eligible participants will be recruited and randomly allocated into four groups using a random number table: the HD-tDCS + CCRT group (Group 1), the HD-tDCS group (Group 2), the CCRT group (Group 3), and the control group (Group 4). Assessments will be carried out at baseline (T0), after 10 sessions (T1), after 20 sessions (T2), and after six months of follow-up (T3). Research framework is illustrated in Fig. 1. Participants in the control group (Group 4) will continue to receive their stable regimen of antipsychotic medication and standard clinical care, without undergoing any HD-tDCS or CCRT sessions.

### 2.2 Recruitment and Eligibility Criteria

This trial will be conducted at the Second Affiliated Hospital of Xinxiang Medical University. Eligible individuals will adhere to the following inclusion criteria: (1) According to the Diagnostic and Statistical Manual of Mental Disorders, Fifth Edition (DSM-5), the individual meets the clinical criteria for schizophrenia. (2) Patients who maintain stability with oral antipsychotic medication are defined by the following criteria: a score of ≤5 on the items of exaggeration, delusion, suspiciousness/victimization, and hallucinatory behavior in the Positive and Negative Symptom Scale (PANSS), and a score of ≤4 on the PANSS conceptual disorganization. (3) Currently, treatment involves atypical antipsychotic drugs, with their equivalent doses calculated using the defined daily dose method. (4) Individuals aged 18 to 50 years. (5) Educated at the primary school level or higher, and able to comprehend and collaborate to finish the trial. Exclusion criteria: (1) Patients are undergoing the acute phase of the disease and cannot cooperate in completing the examination and operational tasks under guidance. (2) Organic brain lesions, intellectual disabilities, or other severe physical illnesses. (3) Currently undergoing other neurostimulation therapies or evidence-based psychotherapy. (4) There are indications related to contraindications for HD-tDCS. (5) There are visual impairments or significant eye diseases, such as color blindness, color weakness, cataracts, etc. (6) Substance abuse and addiction. (7) Women who are pregnant or breastfeeding.

Clinical professionals who are qualified will conduct the evaluation of potential participants using the inclusion and exclusion criteria. Eligible individuals will receive comprehensive verbal and written details about the study’s benefits, risks, and precautions from psychiatrists. Upon consenting to participate, both the participants and their legal guardians are required to provide their signatures on an informed consent form. The trial will be carried out according to the 2013 Standard Protocol Items: Recommendations for Interventional Trials (SPIRIT) statement guideline for trial protocols [49]. See Table 1 and **Supplementary Material**.

**Table 1.** **World Health Organization trial registration data set related to this study**.

Data category	Information
Primary Registry and Trial Identifying Number	ChiCTR2500102731
Date of Registration in Primary Registry	19. May. 2025
Secondary Identifying Numbers	N/A
Source(s) of Monetary or Material Support	The Second Affiliated Hospital of Xinxiang Medical University, Henan Mental Hospital
Primary Sponsor	Yange Wei, MD. Ph.D., The Second Affiliated Hospital of Xinxiang Medical University, Henan Mental Hospital, 207 Qianjin Road, Xinxiang 453002, Henan. China
Secondary Sponsor(s)	N/A
Contact for Public Queries	Shanyuan He, The Second Affiliated Hospital of Xinxiang Medical University, Henan Mental Hospital, 50240101132@stu.xxmu.edu.cn
Contact for Scientific Queries	Yange Wei, MD. Ph.D., The Second Affiliated Hospital of Xinxiang Medical University, Henan Mental Hospital, weiyange@xxmu.edu.cn
Public Title	Efficacy and mechanisms underlying MRI-guided high-definition transcranial direct current stimulation combined with computerized cognitive remediation therapy for improving cognitive impairments in schizophrenia: study protocol for a randomized controlled trial
Scientific Title	Efficacy and mechanisms underlying MRI-guided high-definition transcranial direct current stimulation combined with computerized cognitive remediation therapy for improving cognitive impairments in schizophrenia: study protocol for a randomized controlled trial
Countries of Recruitment	China
Health Condition(s) or Problem(s) Studied	Schizophrenia
Intervention(s)	High-definition transcranial direct current stimulation (HD-tDCS) and/or computerized cognitive remediation therapy (CCRT)
Inclusion and Exclusion Criteria	Inclusion criteria:
	(1) According to Diagnostic and Statistical Manual of Mental Disorders, Fifth Edition (DSM-5), the individual meets the clinical criteria for schizophrenia.
	(2) Patients who maintain stability with oral antipsychotic medication are defined by the following criteria: a score of ≤5 on the items of exaggeration, delusion, suspiciousness/victimization, and hallucinatory behavior in the Positive and Negative Symptom Scale (PANSS), and a score of ≤4 on the PANSS conceptual disorganization.
	(3) Currently, treatment involves atypical antipsychotic drugs, with their equivalent doses calculated using the defined daily dose method.
	(4) Individuals aged 18 to 50 years.
	(5) Educated at the primary school level or higher, and able to comprehend and collaborate to finish the trial.
	Exclusion criteria:
	(1) Patients are in the acute phase of the disease and cannot cooperate in completing the examination and operational tasks under guidance.
	(2) Organic brain lesions, intellectual disabilities, or other severe physical illnesses.
	(3) Currently undergoing other neurostimulation therapies or evidence - based psychotherapy.
	(4) There are indications related to contraindications for HD-tDCS.
	(5) There are visual impairments or significant eye diseases, such as color blindness, color weakness, cataracts, etc.
	(6) Substance abuse and addiction.
	(7) Women who are pregnant or breastfeeding.
Study Type	Interventional
	Allocation: randomized
	Masked: double blind
	Primary purpose: schizophrenia intervention
Date of First Enrollment	June 2025
Sample Size	48
Recruitment Status	Pending
Primary Outcome(s)	(1) MATRICS Consensus Cognitive Battery (MCCB)
	(2) Brief Psychiatric Rating Scale (BPRS)
	(3) Positive and Negative Syndrome Scale (PANSS)
Key Secondary Outcomes	(1) Clinical Global Impressions (CGI)
	(2) Social Disability Screening Schedule (SDSS)
	(3) Schizophrenia Quality of Life Scale (SQLS)
	(4) Magnetic Resonance Imaging (MRI)
	(5) General Information Questionnaire (GIQ)
	(6) Adverse Reaction Scale (ARS)
	(7) Serum levels of Brain-Derived Neurotrophic Factor (BDNF)
	(8) Serum levels of Glial Cell Line-Derived Neurotrophic Factor (GDNF)
Ethics Review	Approved (Approval Number: XYEFYLL-2025-15)
	Approval Date: 17 February 2025
	The Second Affiliated Hospital of Xinxiang Medical University. xyefyll@126.com, +86 0373-3373500
Completion date	Pending
Summary Results	Pending
IPD sharing statement	N/A

The research could be halted under the following conditions: (1) the occurrence of an unexpected medical emergency; (2) the emergence of severe side effects or adverse events that preclude the continuation of the study; or (3) the withdrawal of informed consent by the participant or their family. It is imperative that participants have maintained a stable dose of antipsychotic medication at least two weeks, and that this dosage remains unchanged throughout the study duration. During the therapy, participants will continue to receive their routine daily treatment.

### 2.3 Randomization 

**Fig. 2.**
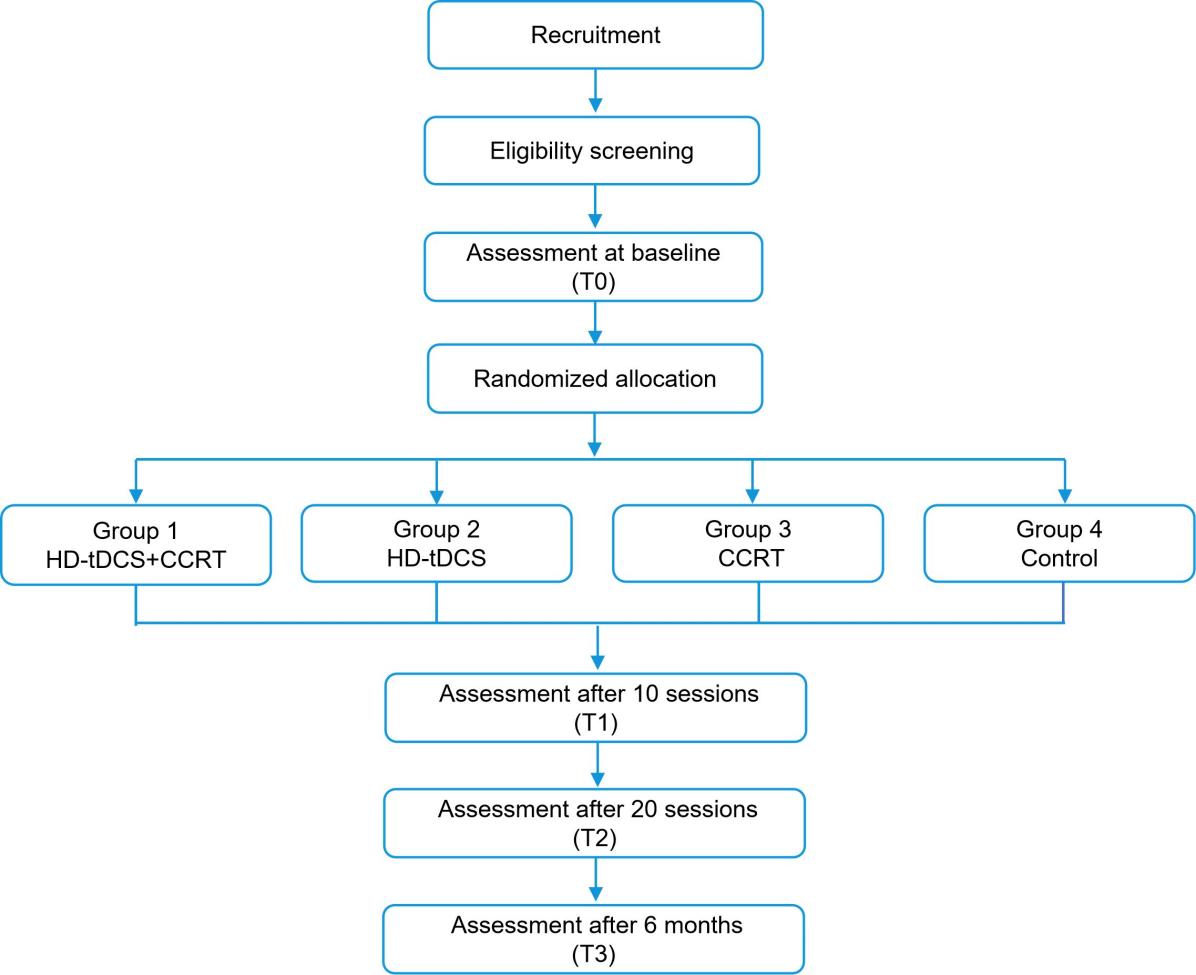
**The flow diagram of the research design**.

Randomization will be performed by a statistician who does not take part in the study. All eligible participants will be allocated into four groups using a computer-generated random number table. Each assigned a specific group number: HD-tDCS + CCRT (Group 1), HD-tDCS (Group 2), CCRT (Group 3), and control (Group 4). For the randomization process, personnel employed Microsoft Excel 2019 software (Microsoft Corp., Redmond, WA, USA) to enter 48 participants into a spreadsheet, assigning each a unique identifier ranging from “1–48”. They then used the “=RAND()” function to generate a random number for each assigned number. Subsequently, these random values were fixed using the “Paste Special” dialog box. Following this, the fixed random values were sorted in descending order. The sorted numbers were then divided into four groups, each comprising 12 numbers, resulting in a total of 48 numbers. This procedure effectively randomized the 48 patients into four groups, with each group consisting of 12 individuals. Once the randomization scheme was established, it was secured in opaque envelopes, which were opened sequentially according to the order of participant enrollment. The allocation scheme within each envelope determined the group assignment for each patient. This method ensured that study personnel could not compromise the randomization process by having prior knowledge of the randomization scheme. The progression of participants through the screening, randomization, therapy, and follow-up stages is illustrated in Fig. 2.

### 2.4 Blinding

Throughout the study period, both participants and evaluating psychiatrists remained blinded. In implementing blinding procedures, we will adhere to these considerations and measures. The specific group assignments of patients will be disclosed only to the principal investigator. Patients were unaware of the details of allocation sequences and were explicitly instructed not to discuss with each other any aspects of group allocation, received therapies, questionnaire completion, or other experimental procedures. Participants will keep unaware of the allocation sequences and will be explicitly instructed not to discuss any aspects of group allocation, receive therapies, questionnaire completion, or other experimental procedures. To minimize potential interference with blinding by therapy personnel, we will employ four distinct therapy groups, each responsible for implementing their respective therapies. Each group was informed only of their own protocol and had no knowledge of group allocation details or the therapy plans of other groups. Communication between groups regarding specific therapy content will be strictly prohibited. During the experimental phase, the principal investigator will provide each group with the specific protocol for their corresponding group, after which each group will implement the therapies for their assigned patients. During the outcome measurement phase, personnel responsible for data collection and analysis will keep blinded to group allocation and specific therapy. The principal investigator implemented a “two-stage unblinding” procedure. Once the data was locked, the initial unblinding was conducted, dividing the data into Groups 1–4 without disclosing their actual relationships or true assignments. The unblinding will be conducted after the data analysis is completed, clearly indicating the specific group representations of 1–4.

### 2.5 MRI Scanning

All participants will receive MRI scans at T0, T1, T2, and T3. MRI scans will be conducted in the Department of Radiology at the Second Affiliated Hospital of Xinxiang Medical University. The scanning procedures will be performed by radiologists who are proficient in MRI operation. A 3.0T MRI scanner (MAGNETOM PRISMA, Siemens Healthineers, Erlangen, Germany) will be used for the scans. Structural T1-weighted MRI images will be acquired using a 3D MPRAGE sequence with the following parameters: Field of View (FOV) = 224 × 224, flip angle = 8 degree, Repetition Time (TR) / Echo Time (TE) / Inversion Time (TI) = 2400/2.14/1000 ms, voxel size = 0.7 mm isotropic, Bandwidth (BW) = 210 Hz/pixel, integrated Parallel Acquisition Techniques (iPAT) = 2. T2-weighted images will be obtained using a 3D T2-SPACE sequence, with the following parameters: TR/TE = 3200/565 ms, flip angle variable, FOV = 224 × 224, voxel size = 0.7 mm isotropic, BW = 755 Hz/pixel, iPAT = 2. Resting-state MRI data will be acquired using a gradient echo planar imaging sequence with the following parameters: TR = 2000 ms, TE = 30 ms, FOV= 220 mm × 220 mm, slice thickness = 4 mm, inter-slice gap = 0.6 mm, number of slices = 33, in-plane resolution = 64 × 64, flip angle = 90°.

### 2.6 MRI-guided High-Definition Transcranial Direct Current Stimulation

MRI scans will be employed to construct detailed anatomically realistic head models. The development and simulation of head models for the HD-tDCS-induced electric field use the SimNIBS software (version 4.5, SimNIBS Group, Copenhagen, Denmark) [30]. Eight distinct 4*1 montages centered on the mPFC will be used to simulate the E-field for each brain. The normal component of the E-field will be calculated using the finite element method [31]. Based on T1 and T2 MRI scans, head models will be constructed from these images, incorporating five different tissues: skin, skull, white matter, gray matter, and CSF. Finally, the mPFC will be identified in each subject’s brain according to the Ranta atlas [32]. The study will utilize an MRI-guided HD-tDCS device (model MxN-9-9002A, Soterix Medical, New York, NY, USA). The central electrode will be positioned over the mPFC. The four surrounding cathodes will be positioned at frontal sites Fp1, Fp2, F7, and F8. The current intensity will be maintained at 2 mA. Each HD-tDCS session will involve the delivery of a 2 mA direct current for a duration of 30 minutes. HD-tDCS will be conducted 5 times per week, over a 4-week period, 20 sessions. During the therapy period, patients will continue their existing pharmacological treatment regimens without any modifications.

### 2.7 Computerized Cognitive Remediation Therapy

This study will employ the CCRT system (Nanjing Weisi Medical Technology Co., Ltd., model VCRT-G, Nanjing, Jiangsu, China) from the Rehabilitation Medicine Department of the Second Affiliated Hospital of Xinxiang Medical University. Patients will receive training under the guidance of trained professional therapists. The hardware architecture of the CCRT system comprises a single server and multiple treatment terminals, while the software architecture is divided into three components: the frontend, backend, and an extensive database. The frontend is integrated with multiple therapy terminals. Upon accessing a terminal, therapists are presented with all available training tasks, enabling patients to engage directly in cognitive training activities. The backend functions as the central host, primarily utilized for entering general patient information, selecting and initiating tasks, and monitoring and reviewing task completion status. The database is responsible for storing all patient-related therapy data, thereby facilitating statistical analyses by researchers. The CCRT includes 6 therapeutic modules: executive function, learning and memory, attention, perceptual-motor skills, language, and social cognition. Treatment is administered sequentially through these modules in a progressive manner. During each session, patients undertake multiple therapeutic tasks derived from various exercises, with each exercise offering multiple levels of difficulty.

The CCRT system encompasses over 10 distinct therapeutic tasks. Below are several examples: (1) Emotion Recognition: Patients observe facial expressions in presented images and select the corresponding emotional label. Correct choices earn points, enhancing social cognition; (2) Voice Coach: Patients listen to audio prompts and drag the described image into a collection box. Higher accuracy and quantity improve scores, targeting language comprehension; (3) Whack-a-Mole: Patients quickly tap moles randomly appearing from holes. Faster responses yield higher scores, training rapid decision-making; (4) Supermarket Shopping: A simulated store scenario where patients follow a shopping list to locate items across sections, integrating executive function training; (5) Train Arrival: Patients adjust track switches to guide color-matched trains to correct stations, refining attentional control; (6) Task Cards: Patients identify and drag cards matching textual instructions into a collector, boosting pattern recognition; (7) Memory Master: Patients memorize sequentially displayed items and recall them under time constraints, strengthening memory retention. The system automatically adjusts the current training difficulty based on indicators such as the patient’s accuracy rate and response time from previous cognitive training stages, thereby adapting to improvements in the patient’s cognitive functions. CCRT is administered once daily, 5 times per week, over a period of 4 weeks, culminating in a total of 20 sessions. Relevant studies indicate that the concurrent application of tDCS with cognitive training enhances task accuracy more effectively than when these therapies are conducted separately [43]. Consequently, it has been determined that in Group 1 (HD-tDCS + CCRT) will be administered simultaneously rather than sequentially.

### 2.8 Blood Sampling and Analysis

**Fig. 3.**
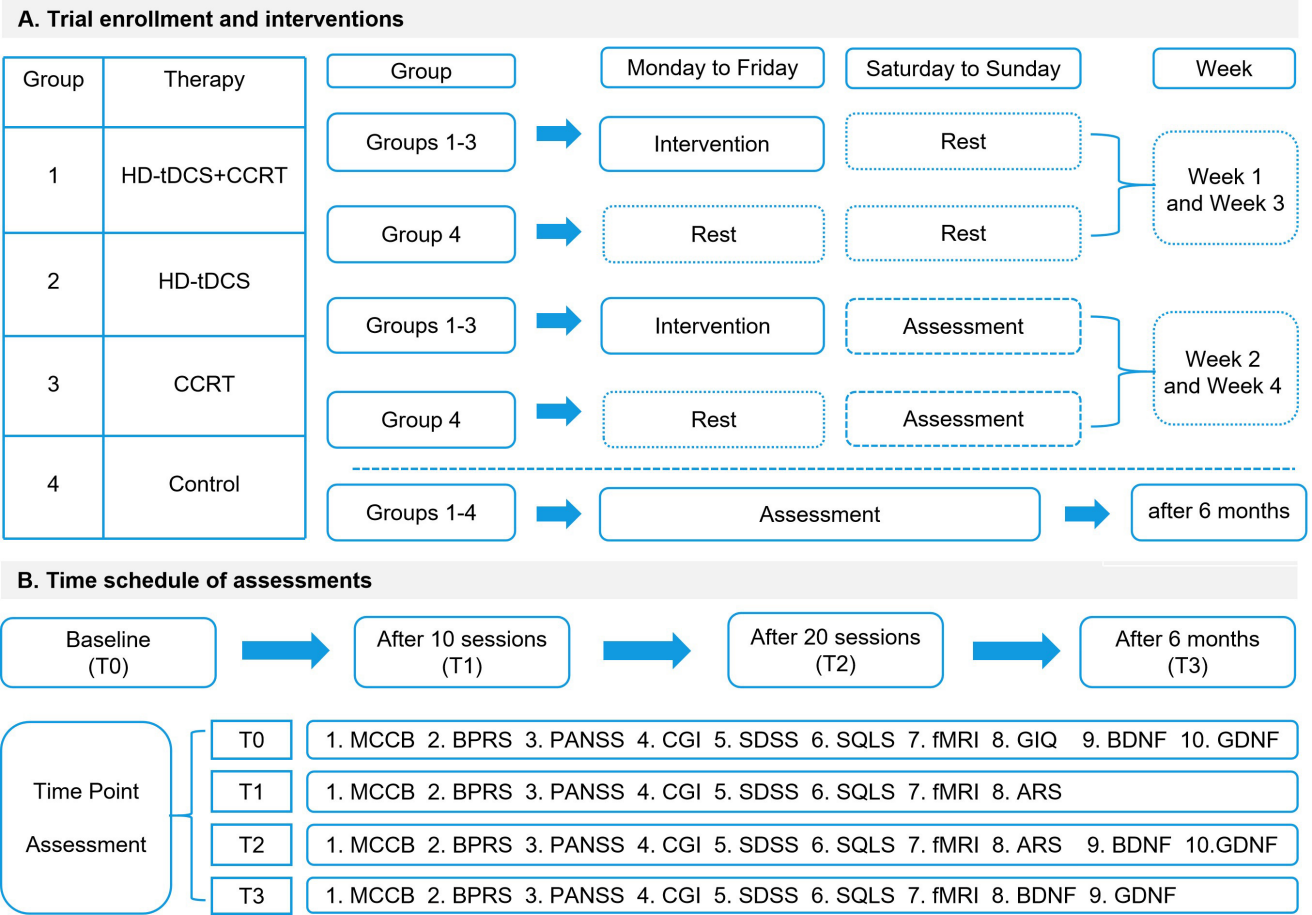
**Schematic representation of the randomized controlled trial**. The experimental timeline, assessment schedule, and grouping arrangements. (A) Introduces the sequence of interventions as well as the timing and content of assessments. (B) Details how intervention sessions will be administered by week. ARS, adverse reaction scale; BDNF, brain-derived neurotrophic factor; BPRS, Brief Psychiatric Rating Scale; CGI, Clinical Global Impressions; fMRI, functional magnetic resonance imaging; GDNF, glial cell line-derived neurotrophic factor; GIQ, General Information Questionnaire; PANSS, Positive and Negative Syndrome Scale; SDSS, Social Dysfunction Screening Scale; SQLS, Schizophrenia Quality of Life Scale.

After a 8-hour fast, 5 mL of blood will be collected into an EDTA-K_2_ anticoagulant tube. Post-centrifugation at 3000 rpm for 10 minutes, two distinct polyethylene tubes will be used to separate the supernatant from the sedimented blood cells. The supernatant and sedimented blood cells will be separated into two distinct polyethylene tubes. The protein concentration in the supernatant will be determined using the BCA kit (Qingdao Jieshikang Biotechnology Co., Ltd., Cat# RYX148, Qingdao, Shandong, China). Subsequently, protein samples and protein molecular weight markers in equal volumes will undergo Tris-sodium dodecyl sulfate polyacrylamide gel electrophoresis. The gel will be moved onto a polyvinylidene fluoride membrane and blocked with a 5% skim milk solution for two hours. Following washing, primary antibodies against BDNF, GDNF, and β-actin (dilution 1:500) will be added and incubated overnight at 4 °C. The antibodies used were as follows: anti-BDNF (Cat# 28205-1-AP, Proteintech Group, Inc., Wuhan, Hubei, China); anti-GDNF (Cat# 26179-1-AP, Proteintech Group, Inc.); and anti-β-actin (Cat# 66009-1-Ig, Proteintech Group, Inc.). The horseradish peroxidase (HRP)-conjugated secondary antibodies to be used are as follows: anti-rabbit IgG (for BDNF and GDNF detection; Cat# SA00001-2, Proteintech Group, Inc.) and anti-mouse IgG (for β-actin detection; Cat# SA00001-1, Proteintech Group, Inc.). Specific lot numbers will be recorded upon procurement. Then, these secondary antibodies will be added at a dilution of 1:5000 and incubated at room temperature for two hours. The PVDF membrane (Cat# E801, Vazyme, Nanjing, Jiangsu, China) containing the target proteins will be placed in a chemiluminescent developer, with the exposure time adjusted according to protein abundance. Quantitative analysis of BDNF and GDNF expression will be conducted using ImageJ software (version 1.53t, National Institutes of Health, Bethesda, MD, USA). Each assay parameter will be measured in duplicate for all samples. The identities of all subjects will be coded by the investigator until the completion of all biochemical analyses. Fig. 3 provides a comprehensive overview of the trial’s timeline, assessment schedule, and arrangements.

### 2.9 Outcome

The primary outcome measure used in this research is the MATRICS Consensus Cognitive Battery (MCCB), which is a crucial tool for assessing cognitive deficits in schizophrenia. It evaluates seven distinct cognitive domains: working memory, abstract thinking, attention/vigilance, processing speed, visual learning, verbal/visual learning, reasoning and problem-solving. The battery comprises the following subtests: (1) Trail Making Test A (TMT-A), (2) Symbol Coding, (3) Hopkins Verbal Learning Test-Revised (HVLT-R), (4) Digit Span Test (DST), (5) Stroop Color-Word Test, (6) Spatial Span Test (derived from the Wechsler Memory Scale III, WMS-III), (7) Verbal Fluency Test (VFT), (8) Mazes, (9) Brief Visuospatial Memory Test-Revised (BVMT-R), and (10) Continuous Performance Test-Identical Pairs (CPT-IP).

Secondary outcome measures include the Brief Psychiatric Rating Scale (BPRS), the PANSS, the Clinical Global Impression (CGI) scale, the Social Disability Screening Schedule (SDSS), the Schizophrenia Quality of Life Scale (SQLS), and fMRI. Specifically, the general information questionnaire is utilized to collect basic information about the patients. BPRS primarily assesses the severity of psychotic symptoms, with total scores ranging from 18 to 126, where higher scores reflect more severe symptoms. PANSS is used to evaluate the presence and severity of various symptoms. CGI scale is applied to assess the patient’s overall condition and therapeutic efficacy. SDSS measures the extent of social functional deficits. SQLS is designed to evaluate the quality of life in schizophrenia. For detailed information, please see Table 2.

**Table 2.** **Schedule of enrollment, interventions, assessments, and visits of patients**.

	Recruitment	Baseline	Assessments		
			Weekend 2	Weekend 4	6-month follow-up
Time point		T0	T1	T2	T3
Prescreening for eligibility, consenting and clinical interview					
Recruitment					
Eligibility screening	√				
Informed consent	√				
Allocation	√				
Primary outcome assessment					
MCCB		√	√	√	√
Second outcome assessment					
BPRS		√	√	√	√
PANSS		√	√	√	√
CGI		√	√	√	√
SDSS		√	√	√	√
SQLS		√	√	√	√
GIQ		√			
MRI		√	√	√	√
Blood sample collection (BDNF and GDNF levels)		√		√	√
Safety					
ARS			√	√	

A checkmark (√) indicates the time point at which each assessment is carried out. MRI, magnetic resonance imaging.

### 2.10 Data Collection and Management

The assessment and examination related to the study scales will be conducted by clinical physicians who are external to the experiment and remain blinded to the study content. Outcome data collection will be managed by dedicated personnel, also external to the experiment, and will be systematically recorded in electronic Case Report Forms (CRFs). To ensure patient confidentiality, patient identification numbers will be utilized during data entry. The collected data will be transferred to a secure database for storage. After all data collection is completed, specialized analysts will conduct. Access to the data will be restricted exclusively to the principal investigator, data collectors, and analysts, and no modifications or exports will be permitted without justification. As patients will undergo follow-up and re-evaluation six months post therapy, they will be contacted and reminded via telephone, email, social media, and other communication methods at the appropriate time. During this period, no one except the principal investigator, data collectors, and analysts will be allowed to access, view, modify, or export the data without cause. Since patients will be followed up and re-evaluated six months after the ends, we will contact and remind them via phone calls, emails, social media, and other means at that time. During the data collection phase, participants who report significant changes in their antipsychotic medication regimen within the 6-month follow-up period will be identified and documented. Data from these participants will be included in all analyses up to the T2 time point, but will be excluded from the primary analysis of the T3 (6-month follow-up) data to prevent confounding the primary study outcomes.

### 2.11 Statistical Analysis Plan

The sample size was calculated using G*Power software (version 3.1.9.7, Heinrich-Heine-Universität Düsseldorf, Düsseldorf, Germany), employing an F-test and a repeated-measures ANOVA model. In this study, we established the probability of a Type I error (α) at 0.05, the probability of a Type II error (β) at 0.20, and the statistical power (1 - β) at 0.80, with an effect size (f) of 0.25. The required sample size was calculated to be 36 participants. Accounting for a 20% dropout rate and ensuring equal group sizes, the target sample size was determined to be 48 participants, with 12 participants per group. The calculation of the sample size mentioned above is based on the sample size calculation methods adopted in previous literature [50]. An article suggests that when designing a preliminary study with no prior information for reference, a sample size of 12 participants per group is appropriate based on feasibility, improvements in the precision of mean and variance estimates, and regulatory considerations [51]. The sample size in this study satisfies the previously mentioned criteria and will offer enough statistical power to achieve the objectives.

We will utilize histograms and the Shapiro-Wilk test to assess the normality of the data distribution. For normal distribution data, continuous variables will be reported as mean ± standard deviation, whereas categorical data will be presented as frequencies (n) and percentages (%). Statistical tests such as one-way analysis of variance (ANOVA), Mann-Whitney U test, *t*-test, and chi-square test will be employed to assess baseline differences among groups. In accordance with the Consolidated Standards of Reporting Trials (CONSORT) guidelines, all analyses will be conducted using the intention-to-treat (ITT) principle, whereby participants will be analyzed according to their original randomization group. To address missing data within the ITT framework, we will employ linear mixed-effects models. These models are appropriate for making valid inferences under the missing-at-random assumption and are well-suited for managing the unbalanced data resulting from participant drop-out. To specifically assess the synergistic effect between HD-tDCS and CCRT, the models will incorporate the HD-tDCS × CCRT interaction as a fixed effect. We will conduct sensitivity analyses by comparing the results derived from mixed-effects models to evaluate the robustness of our findings. Line charts will be employed to visualize the data, offering a clear depiction of outcome value trends across time points. Given the repeated-measures nature of the data, we will utilize statistical methods suitable for this design. In addition to the mixed-effects models, we will explore non-parametric repeated-measures approaches where applicable. These methods are suitable for analyzing between-group differences at specific time points and changes within groups over time for both primary and secondary outcomes. Moreover, the Likelihood Ratio Test will be used to specifically assess the effect of the therapy. The Bonferroni correction will be employed to adjust *p*-values, addressing the issue of multiple comparisons from evaluations at different time points.

To further elucidate the underlying mechanisms, we will conduct correlation analyses to investigate the relationships between alterations in neuroimaging metrics, serum levels of BDNF/GDNF, and cognitive performance scores in participants. Additionally, we intend to utilize mediation analysis models to assess whether intervention-induced changes in brain activity and/or BDNF/GDNF levels mediate improvements in cognitive outcomes. Moreover, we will perform an exploratory analysis of the CCRT metrics (e.g., task accuracy, reaction time) to compare cognitive performance during training between the HD-tDCS + CCRT group (Group 1) and the CCRT group (Group 3). This analysis could clarify the immediate neuroenhancing potential of concurrent HD-tDCS.

A stable antipsychotic medication regimen will be required for all patients, requiring consistent dosing for a minimum of two weeks prior to the trial and maintaining this regimen unchanged throughout the study period. This protocol will be implemented to minimize confounding effects on clinical outcomes, in accordance with recommendations from previous literature [52]. The Defined Daily Dose (DDD) methodology will be utilized to standardize antipsychotic medication doses across study groups. This involves converting administered doses into DDD units following randomization to ensure pharmacological equivalence. To account for dosing variability and maintain the accuracy of results, DDD values will be incorporated as a covariate in all statistical models. All collected data will be entered into a database and analyzed using SPSS version 20.0 (IBM Corp., Chicago, IL, USA). Significance is defined as *p* < 0.05.

### 2.12 Data Monitoring

Given the minimal risk associated with the study, the Institutional Review Board of the Second Affiliated Hospital of Xinxiang Medical University waived the requirement for a data monitoring committee.

### 2.13 Harms

To examine the safety of HD-tDCS and track any adverse reactions participants might experience, an adverse reaction scale will be employed. An adverse reaction assessment form specific to HD-tDCS was developed for this study, encompassing common adverse reactions such as headache, skin tingling, fatigue, itching, burning sensations, and local discomfort. The design of this scale facilitates the prompt identification and management of any issues that may arise during the process. In the event of a serious adverse reaction, the current therapy will be discontinued, appropriate health evaluations will be conducted, and the incident will be communicated to the ethics committee. All adverse events will be systematically recorded in the CRF with respect to their incidence, types, and severity. The final study results will include a comprehensive summary of adverse events for each study group (Groups 1–4), including the frequency, severity, and percentage of participants affected by each type of adverse event. This will enable safety comparisons across the different interventions. To mitigate potential adverse reactions associated with the study, professional therapists will be engaged to implement the therapy, and they will receive comprehensive training pertinent to the study.

### 2.14 Auditing

To ensure data integrity and consistency, the principal investigator will conduct weekly reviews of the CRFs and complete the estimation forms. To maintain consistency, research records in both paper and digital formats will be verified every two weeks. Any discrepancies identified will be thoroughly documented and discussed during research team meetings to facilitate timely corrective actions. These checks will be conducted throughout the entire study period, including during the therapy and 6-month follow-up assessment period.

### 2.15 Protocol Modifications

All significant amendments to the study protocol will first be presented to the Ethics Review Committee of the Second Affiliated Hospital of Xinxiang Medical University. Once approved, these changes will be documented in writing to all relevant parties and properly recorded on the Chinese Clinical Trial Registry platform.

### 2.16 Dissemination Policy

Before the first patient is enrolled in the trial, relevant trial information will be published on the website of the Chinese Clinical Trial Registry. We are devoted to making all research findings accessible to the public. These findings will not only be presented and deliberated at international conferences but also submitted for publication in peer-reviewed academic journals.

## 3. Expected Results

This study will evaluate between-group differences at specific time points and within-group changes over time for both primary and secondary outcomes. We hypothesize that MRI-guided HD-tDCS combined with CCRT will result in more significant improvements compared to those receiving HD-tDCS or CCRT alone. Following four consecutive weeks of treatment, it is anticipated that the combined group will exhibit amelioration of prefrontal dysfunction, as measured by MRI, and demonstrate significantly elevated expression levels of BDNF and GDNF.

## 4. Discussion

To the best of our knowledge, this prospective, randomized, double-blind, controlled trial represents the first to investigate the synergistic effects of MRI-guided HD-tDCS targeting mPFC and CCRT on cognitive dysfunction in schizophrenia. First, we will employ MRI-guided HD-tDCS to ensure that the electrical current is confined to the targeted region of the mPFC, delivering more precise and focused stimulation. Further, we present a randomized, double-blind, controlled design to investigate the potential role of cognitive improvement in schizophrenia through the MRI-guided HD-tDCS and CCRT. Second, cognitive impairment is the core symptom of schizophrenia, which not only severely affects the overall quality of life of patients but also places significant burdens on their caregivers [53]. Herein, this study will evaluate not only the short-term effects in schizophrenia, but also address the long term effects after six months of follow-up. Third, multiple objective methods will contribute to a comprehensive understanding of the mechanisms of HD-tDCS and CCRT in schizophrenia. We hypothesize that this combined group will exhibit more pronounced improvements compared to those receiving either HD-tDCS or CCRT alone. If confirmed, the study will introduce an efficient method for addressing cognitive impairments, thereby assisting clinical psychiatrists in selecting individualized therapy strategies and potentially improving patients’ quality of life and social functioning.

In this study, we propose MRI-guided HD-tDCS targeting the mPFC to ameliorate cognitive deficits in patients with schizophrenia. The mPFC is intrinsically associated with memory and decision-making functions in humans [54]. Individuals with schizophrenia often exhibit pronounced functional abnormalities in the mPFC, which may impair their cognitive control capabilities and lead to reduced performance on complex tasks [55]. Research indicates that HD-tDCS targeting the mPFC can enhance activation in the insula while decreasing activation in the amygdala, resulting in improvements in attention, working memory, and probabilistic learning [56]. Accordingly, HD-tDCS targeting the mPFC has been shown to enhance decision-making behaviors under conditions of low outcome controllability, thereby promoting more adaptive behavioral performance [57]. The specific mechanisms of tDCS involve the activation of N-methyl-D-aspartate receptors and the upregulation of BDNF expression, both of which contribute to the enhancement of synaptic plasticity. Additionally, tDCS influences the release of neurotransmitters. The modulation of glutamate and glutamine release can influence neuronal excitability, thereby facilitating the processes of learning and memory formation [58, 59, 60]. This modulation not only contributes to short-term cognitive improvements but may also lead to long-term cognitive enhancements through the mechanism of long-term potentiation (LTP) [55]. Consequently, MRI-guided HD-tDCS targeting mPFC emerges as a promising and precise strategy for enhancing cognitive function at an individual level.

Interestingly, CCRT has been shown to ameliorate cognitive impairments by modulating neural activity within the mPFC [61]. CCRT has been observed to increase brain activity in the mPFC, parietal cortex, insula, caudate nucleus, and thalamus [62]. Recent studies have demonstrated that following CCRT, the amplitude of low-frequency fluctuations (ALFF) in the mPFC was significantly increased, while ALFF in the brainstem was significantly decreased in patients with schizophrenia [63]. Additionally, neuroimaging studies have revealed that CCRT may enhance cognitive function in schizophrenia by increasing the integrity of the anterior corpus callosum white matter fibers, thereby improving the efficiency of information transfer in the bilateral mPFC [64]. CCRT has been shown to significantly elevate GDNF levels in patients [42]. These findings indicate that CCRT may facilitate cognitive enhancement by modulating neural activity in the mPFC and regulating neurotrophic factor levels. In conclusion, CCRT presents a potentially effective therapeutic strategy for enhancing cognitive function in individuals with schizophrenia through computerized, individualized training and associated mechanisms.

The integration of HD-tDCS and CCRT offers a valuable adjunct to conventional antipsychotic therapies. Patients with schizophrenia often require prolonged use of antipsychotic medications, which can result in side effects such as extrapyramidal symptoms, endocrine disturbances, and metabolic issues. By reducing dependence on pharmacological therapies, HD-tDCS and CCRT may help mitigate these adverse effects, thereby enhancing patients’ overall quality of life. Additionally, a notable advantage of this combination is its convenience and cost-effectiveness. HD-tDCS devices are relatively portable and user-friendly, rendering them suitable for extensive application use in home settings. This methodology is supported by a recent quantitative summary confirming the efficacy, safety, and tolerability of home-based tDCS [65]. CCRT content can be delivered via online platforms, enabling patients to manage their treatment independently at home and thereby minimizing the necessity for frequent hospital visits. Consequently, the integration of HD-tDCS with CCRT not only offers economic advantages but also enhances the convenience of treatment. This synergy is likely to improve patient adherence and increase the flexibility of therapeutic interventions. Due to its affordability and accessibility, this combined approach is well-suited for community health centers, rehabilitation facilities, and home-based care. Future studies should focus on determining the optimal parameters for HD-tDCS and CCRT, such as current intensity, stimulation duration, and specific CCRT task types, as well as the development of standardized protocols for their implementation in clinical practice.

More importantly, this study will utilize structural and functional MRI to assess the modulatory effects of HD-tDCS in conjunction with CCRT on brain activity in individuals with schizophrenia. By measuring blood oxygen level-dependent signals, fMRI provides high spatial resolution insights into neural activity changes in specific brain regions and elucidates the dynamic interactions within large-scale brain networks. This imaging technique facilitates the observation of neural activity in targeted brain regions, enabling correlations with cognitive functions and predictions of therapeutic outcomes. Interestingly, the mPFC, part of the default mode network, shows increased activity in patients during working memory tasks [66]. Previous fMRI studies examining spontaneous neural activity have demonstrated that functional connectivity between the mPFC and the left orbitofrontal cortex in schizophrenia is positively correlated with performance on the DST and other cognitive assessments [67]. We hypothesize that the combined approach will result in altered activity within the mPFC, potentially correlating with cognitive improvements. Collectively, multimodal MRI approaches have deepened our understanding of the possible mechanisms of HD-tDCS and CCRT in schizophrenia, which is essential for precise medicine with significantly improved outcomes.

This study also focuses on the potential role of BDNF and GDNF in enhancing cognitive function through the combined application. BDNF, the most ubiquitously distributed neurotrophic factor in the brain, is integral to synaptic plasticity and learning processes, with its increased expression positively affecting LTP and memory formation [68, 69]. In patients with schizophrenia, plasma BDNF levels have been shown to change in parallel with cerebrospinal fluid BDNF levels, indicating that peripheral measurements may serve as reliable markers of central BDNF status [43]. Furthermore, studies have shown reduced BDNF levels in individuals with schizophrenia, which tend to increase following therapeutic interventions [40]. GDNF is essential for the nervous system, as it supports the survival, differentiation, and function of dopaminergic neurons, thereby maintaining normal brain activity [70]. A meta-analysis has demonstrated significantly lower GDNF levels in patients compared with healthy controls, with antipsychotic medication restoring these levels [71]. Based on these findings, it has been suggested that BDNF and GDNF may serve as potential biomarkers and predictors of therapeutic response in schizophrenia. Monitoring GDNF levels may facilitate the optimization of personalized therapeutic strategies for schizophrenia. Consequently, this study aims to examine serum alterations in BDNF/GDNF pre- and post-intervention to investigate their potential role in mediating cognitive improvements, thereby providing biological evidence for the combined therapy.

It is important to note that variables such as illness duration and long-term antipsychotic treatment may influence treatment efficacy. Research suggests that chronic antipsychotic use can induce adaptations in dopaminergic pathways, including upregulation of dopamine transporters, decreased firing rates of dopamine neurons, and decreased synaptic vesicle release [72]. Additionally, antipsychotics may affect synaptic function by modifying the levels of postsynaptic density protein 95 and altering dendritic spine density [73]. A longitudinal study investigating first-episode schizophrenia patients revealed that after two years of pharmacological treatment, there were observed increases in basal ganglia and white matter volumes, accompanied by widespread cortical thinning [74]. Individuals with chronic, untreated schizophrenia exhibit more pronounced progressive deterioration of brain structures—such as reductions in gray matter volume, diminished white matter integrity, and disrupted brain network connectivity—compared to those who have been recently diagnosed or treated [75]. These findings suggest that the complex interplay between the pathophysiological processes of the disease and its pharmacological management may influence the therapeutic efficacy of both HD-tDCS and CCRT. Future research should incorporate subgroup analyses based on illness duration to specifically investigate how these factors may moderate the interaction between HD-tDCS and CCRT.

This study still has some limitations. Firstly, the relatively small sample size might influence the statistical power and limit the generalizability of the outcomes. Future research should consider increasing the sample size to enhance statistical robustness. Secondly, the absence of a double-sham control group, which would receive both sham HD-tDCS and a sham version of CCRT, complicates the ability to definitively differentiate between specific therapeutic effects and potential placebo effects. Additionally, the lack of corresponding sham controls for the HD-tDCS-alone and CCRT-alone groups constrains our capacity to accurately assess the independent contributions of each monotherapy. Thirdly, we intend to collect resting-state fMRI data, which could not provide task-specific information. Future study will employ task state fMRI to reflect the fMRI images of the brain when performing specific tasks. Finally, the mechanisms underlying the combined therapy remain relatively unexplored, particularly regarding how these two modalities synergistically enhance cognitive function through specific pathways. This necessitates further comprehensive research to elucidate these mechanisms.

This study protocol establishes an important theoretical framework for exploring the combined therapy and provides guidance for future research. Using MRI-guided HD-tDCS targeting mPFC will enhance the development of individualized therapies for schizophrenia. Findings from multimodal neuroimaging and blood biomarkers could uncover the mechanisms of combining MRI-guided HD-tDCS with CCRT for schizophrenia and contribute to develop more individualized approaches. As a result, this integrated therapeutic strategy holds significant promise for translation into clinical practice.

## 5. Conclusion

In summary, this protocol outlines the first randomized controlled trial designed to evaluate the synergistic efficacy and underlying mechanisms of MRI-guided, mPFC-targeted HD-tDCS combined with CCRT for cognitive impairment in schizophrenia. The study aims to determine whether the combined application of neuromodulation and cognitive training yields superior and more sustained cognitive improvements compared to either intervention alone. The integration of neuroimaging and blood biomarker analyses may help elucidate the associated mechanisms at the imaging and molecular levels. If proven effective, this integrated therapeutic strategy holds potential for translation into clinical practice, offering a new non-pharmacological avenue to address cognitive rehabilitation needs in schizophrenia.

## Availability of Data and Materials

Not applicable.

## Supplementary Material

Supplementary material associated with this article can be found, in the online version, at https://doi.org/10.31083/AP46768.
