# Supplementary material for: Efficacy and Mechanisms Underlying MRI-guided High-definition Transcranial Direct Current Stimulation Combined With Computerized Cognitive Remediation Therapy for Improving Cognitive Impairments in Schizophrenia: Study Protocol for a Randomized Controlled Trial
Source: Alpha Psychiatry. 2026 Feb 2;27(1):46768. doi: 10.31083/AP46768 (PMC12957961; doi:10.31083/AP46768)
Supplement: Supplementary file 1 [file 2757-8038-27-1-46768-s1.zip › Supplementary Material.doc]

**Supplementary Material**

**Efficacy and mechanisms underlying MRI-guided high-definition transcranial direct current stimulation combined with computerized cognitive remediation therapy for improving cognitive impairments in schizophrenia: study protocol for a randomized controlled trial**

**Informed Consent Form**

**1. Introduce**

Schizophrenia is a chronic and severe psychiatric disorder that commonly presents with hallucinations, delusions, emotional blunting, reduced motivation, and cognitive impairments such as deficits in attention, memory, and executive function. Currently available medications have limited efficacy in improving these cognitive symptoms. We would like to introduce a novel non-pharmacological combination intervention: high-definition transcranial direct current stimulation (HD-tDCS) paired with computerized cognitive remediation therapy (CCRT). HD-tDCS is a non-invasive brain stimulation technique that delivers a weak electrical current through the scalp to modulate brain activity, while CCRT uses computerized training programs to enhance cognitive abilities like attention and memory. The combination of these two approaches may help improve cognitive function.

2. **The name of the study and approval for its implementation**

The title of this study is: "Efficacy and mechanisms underlying high-definition transcranial direct current stimulation combined with computerized cognitive remediation therapy for improving cognitive impairments in schizophrenia: study protocol for a randomized controlled trial."

This study has been approved by the Research Ethics Committee of the Second Affiliated Hospital of Xinxiang Medical University (Approval Code: XYEFYLL-2025-15, Approval Date: 17 February 2025) and registered with the Chinese Clinical Trial Registry (Registration Number: ChiCTR2500102731).

3. **Objective**

The primary objective of this study is to evaluate participants’ cognitive function—using the Chinese version of the MATRICS Consensus Cognitive Battery (MCCB)—after they complete 20 sessions of HD-tDCS and/or CCRT. At the same time we will assess psychotic symptoms, overall treatment efficacy, social functioning, quality of life, and related indicators through appropriate scales and examinations. If the treatment proves effective, this research will provide a new therapeutic reference for the treatment of patients with schizophrenia.

4. **High-definition transcranial direct current stimulation**

HD-tDCS is a non-invasive brain stimulation technique that uses a 4×1 electrode array—one small central anode surrounded by four small return electrodes—to deliver a constant, low-intensity current (2 mA in this study) and has shown beneficial effects on psychiatric symptoms and cognitive function.

5. **Computerized cognitive remediation therapy**

CCRT is a computer-based intervention that targets cognitive deficits through digital training. It moves traditional face-to-face cognitive remediation onto a computer platform, using gamified, adaptive exercises to repeatedly engage domains such as attention, executive function, and social cognition, with the ultimate goal of improving patients’ daily functioning and quality of life.

6. **Inclusion and exclusion criteria**
   6.1 **Who will be included?**
(1) Meets the clinical diagnostic criteria for schizophrenia according to DSM-5;
(2) Patients who achieve a stable period through oral antipsychotic drug treatment are defined by the following criteria: a score of ≤5 on the items of delusion, hallucinatory behavior, exaggeration, and suspiciousness/victimization in the Positive and Negative Symptom Scale (PANSS), and a score of ≤ 4 on the PANSS conceptual disorganization;
(3) Current treatment with atypical antipsychotic medications, with equivalent doses of antipsychotic drugs calculated using the defined daily dose method;
(4) Ages between 18-50 years;

(5) Educational level at the primary school level or above, capable of understanding and cooperating to complete the trial.

**6.2** **Who will be excluded?**

(1) Patients are in the acute phase of the disease and cannot cooperate in completing the examination and operational tasks under guidance;

(2) Organic brain lesions, intellectual disabilities, or other severe physical illnesses;

(3) Currently undergoing other neurostimulation therapies or evidence-based psychotherapy;

(4) There are indications related to contraindications for HD-tDCS;

(5) There are visual impairments or significant eye diseases, such as color blindness, color weakness, cataracts, etc;

(6) Severe drug and alcohol dependence;

(7) Pregnant or lactating women.

7. **Intervention plan**
   7.1 **HD-tDCS plan**

You will receive HD-tDCS five times per week for four weeks, once daily, with each session lasting 30 minutes at a current intensity of 2 mA, while continuing your regular medication. The central electrode will be positioned over the mPFC, and the four surrounding cathodes will be placed at the frontal sites Fp1, Fp2, F7, and F8.

7.2 **CCRT plan**

CCRT will be carried out under the supervision of trained therapists. The programme contains six modules—attention, executive function, learning and memory, language, perceptual-motor skills, and social cognition. Sessions will last 30 min and will be run once daily, five days per week, for 4 weeks (20 sessions in total). For participants in Group 1 (HD-tDCS + CCRT), HD-tDCS and CCRT will be delivered concurrently rather than sequentially.

7.3 **control plan**

If you are assigned to the control group (Group 4), you will continue with your daily medication without any intervention of HD-tDCS or CCRT.

The following is how the study will be administered.

1) Firstly, you will be screened to determine if you meet the inclusion criteria and do not meet the exclusion criteria.

2) Once confirmed to participate, you will be randomly assigned to the HD-tDCS + CCRT group (Group 1), the HD-tDCS group (Group 2), the CCRT group (Group 3), and control group (Group 4). The assessors conducting the evaluations and yourself will not know which group you belong to until the study is completed. Before the first stimulation, we will collect your general demographic data and conduct a baseline assessment.

3) The intervention therapy will be carried out according to the plan of the assigned group.

During the study, you will need to report any adverse reactions.

4) You will be reassessed after 10 sessions (T1), after 20 sessions (T2), and after six months of follow-up (T3).

Please note that the study will be discontinued if any of the following conditions occur: (1) the occurrence of an unexpected medical emergency; (2) the emergence of severe side effects or adverse events that preclude the continuation of the study; or (3) the withdrawal of informed consent by the participant or their family.

**Figure 1.** **Schematic overview of the study procedure.**

This figure provides an overview of the experimental workflow and the various components designed for the study.CCRT: Computerized Cognitive Remediation Therapy; HD-tDCS: High-Definition transcranial Direct Current Stimulation；MCCB：MATRICS Consensus Cognitive Battery；MRI：Magnetic Resonance Imaging. T0: baseline; T1: after 10 sessions; T2: after 20 sessions; T3: after 6 months of follow-up.

**Figure 2. Flowchart of the study design.**

This figure illustrates the overall flow of the study design, presented primarily in terms of the sequential order. CCRT: Computerized Cognitive Remediation Therapy; HD-tDCS: High-Definition transcranial Direct Current Stimulation. T0: baseline; T1: after 10 sessions; T2: after 20 sessions; T3: after 6 months of follow-up.

**Figure 3. Schematic representation of the randomized controlled trial.**

This figure outlines the experimental timeline, assessment schedule, and group assignments.

(A) depicts the sequential order in which the interventions are delivered, together with the timing and content of each assessment. (B) specifies how the intervention sessions are conducted on a week-by-week basis. ARS: Adverse Reaction Scale; BDNF: Brain-Derived Neurotrophic Factor; BPRS: Brief Psychiatric Rating Scale; CCRT: Computerized Cognitive Remediation Therapy; CGI: Clinical Global Impressions; fMRI: Functional Magnetic Resonance Imaging; GDNF: Glial Cell Line-Derived Neurotrophic Factor; GIQ: General Information Questionnaire; HD-tDCS: High-Definition transcranial Direct Current Stimulation; MCCB: MATRICS Consensus Cognitive Battery; PANSS: Positive and Negative Syndrome Scale; SDSS: Social Dysfunction Screening Scale; SQLS: Schizophrenia Quality of Life Scale; T0: baseline; T1: after 10 sessions; T2: after 20 sessions; T3: after 6 months of follow-up.

7.4 **Outcome Assessment**

**Primary outcome**

Cognitive function will be assessed before the first stimulation (T0), after 10 sessions (T1), after 20 sessions (T2), and after six months of follow-up (T3) using the Chinese version of the MATRICS Consensus Cognitive Battery (MCCB). The MCCB is an operational and widely used tool for assessing cognitive function, and the evaluator will instruct you on how to proceed. Here is a description of each test:

Trail Making Test A (TMT-A): In this test, participants are required to connect a series of numbers from 1 to 25 that are randomly arranged on a piece of paper in sequential order. Before starting the test, participants will be instructed to complete the connections as quick.

Symbol Coding: In this test, the top of the test sheet lists nine numbers from 1 to 9, each matched with a unique symbol. The task of the participant is to fill in the corresponding blank with the number that matches each symbol appearing below within a 90-second time limit. The number of correct numbers filled in by the participant will serve as their test score.

Hopkin Verbal Learning Test - Revised (HVLT-R): In this test, the examiner will clearly read out 12 nouns belonging to different categories, one word per second. After the reading is finished, the participant is required to attempt to recall and repeat these nouns. This process will be repeated three times, with recall attempts following each reading. The participant earns 1 point for each noun correctly recalled, and no additional points are given for nouns recalled more than once. The scores from the three trials are cumulative, with a maximum possible score of 36 points.

Digit Span Test (DST): This test is divided into two parts: forward recall and backward recall. The test starts with lower digits and gradually increases to higher digits. The examiner reads the digits slowly and clearly, and then the participant is asked to recall and repeat them. If the participant makes two consecutive errors, the test ends. The score is based on the number of correct repetitions, and the scores for forward and backward recall are added together to get the total score.

Stroop Color-Word Test: The test consists of three cards. Card A has the names of colors (red, green, blue, yellow) printed in black ink, and within 60 seconds, the participant is required to read out the names of the colors as quickly and accurately as possible; Card B has the symbol "X" printed in red, green, blue, and yellow colored inks, and the participant is asked to read out the color of each symbol as quickly and accurately as possible within 60 seconds; Card C has the color names (red, green, blue, yellow) randomly printed in red, green, blue, and yellow colored inks, with the color names not matching the ink colors, and the participant is required to read out the color of the ink as quickly and accurately as possible within 60 seconds, disregarding the actual text. The number of correct responses given by the participant within 60 seconds is recorded as the scoring standard for each card.

Wechsler Memory Scale III (WMS III) Spatial Span Test: Divided into forward tapping and backward tapping sections, the test involves presenting a wooden block with no numbers facing the participant. The examiner taps the blocks at a rate of one per second and then asks the participant to recall and tap the blocks in the same order. Each item in the test is subjected to two different trials. Regardless of the outcome of the first trial, the second trial must be conducted. If a participant scores 0 on both the first and second trials for any item, the test is terminated, and the participant proceeds to the backward tapping section, with scoring rules the same as for the forward tapping. A perfect sequence earns 1 point. A completely correct tap once earns 1 point. The highest possible score for the forward sequence test is 16 points, for the backward sequence test is 16 points, and the total highest possible score is 32 points.

Verbal Fluency Test (VFT): Time-limited to 60 seconds, participants are required to name as many animal names as they can think of, and the total score is based on the number of correctly named animals. The scoring rules are as follows: repetitions are not scored; names such as "zebra," "Akhal-Teke horse," and "horse" can all be scored without restrictions on their classification; fabricated animals that have never existed, such as "dragons," are not scored; extinct species that once existed, such as "dinosaurs," can be scored; when nouns are modified by adjectives like colors, only the first use can be scored.

Mazes: The maze test is divided into parts A-G, with each maze having only one entrance and one exit. The route drawn from the entrance to the exit must follow certain requirements: shortcuts at corners are not allowed, and the line must not intersect with the existing segments. As with the TMT-A, the pen should not be lifted from the paper. Of course, you can plan with your hand before starting, but this is included in the timing. If completed within the specified time, points are scored; if not, 0 points are given. If a participant scores 0 points for three consecutive times, the test ends. The scores of each maze are added together to get the total score.

Brief Visuospatial Memory Test - Revised (BVMT-R): On a single picture, there are six geometric figures. Participants are given 10 seconds to observe them and then asked to recall and draw them. If the figure and its position are both correct, 2 points are awarded. If either the figure or the position is incorrect, 1 point is given. If no figure is drawn or there is no similar figure, 0 points are awarded. The entire test process is repeated three times, and the scores from the three attempts are summed to give the total score, with a maximum possible score of 36 points.

Continuous Performance Test - Identical Pair (CPT-IP): Different numbers appear randomly on the computer screen, with two-digit, three-digit, and four-digit numbers forming three sets, each containing 150 numbers, appearing randomly on the computer screen. Participants are instructed to click the left mouse button when they see the same number appear, and the computer automatically calculates and analyzes the score.

**Second outcome**

The following are additional scales and assessments used to evaluate other aspects. Brief Psychiatric Rating Scale (BPRS): Mainly used to assess the severity of patients' psychotic symptoms. Positive and Negative Symptom Scale (PANSS): Assesses the presence and severity of various psychiatric symptoms. Clinical Global Impressions (CGI) Scale: A tool used to assess the condition and therapeutic response of patients. Social Dysfunction Screening Scale(SDSS): A tool for assessing individual social dysfunction. Schizophrenia Quality of Life Scale(SQLS): Used to assess the quality of life of patients with schizophrenia. Functional Magnetic Resonance Imaging(fMRI): Measures the concentration changes of oxygenated and deoxygenated hemoglobin in the cerebral cortex, thereby indirectly reflecting the cerebral blood flow dynamics and neural activity. General Information Questionnaire: Used to collect basic information about participants. Adverse Reaction Scale: Assists researchers and healthcare professionals in rapidly identifying and managing potential problems that may occur during the treatment process, ensuring the safety of the therapy.

8 **What do you need to note?**
a. Provide accurate information about your medical history and current health status.
b. Inform the research physician of any discomfort you experience during this study.
c. Inform the research physician if you have participated in any other studies recently or are currently involved in other research.
d. Please adhere to the corresponding requirements in the study (such as not discussing with other participants what interventions you have undergone, etc.).
9 **The risk and burdens**

You may encounter the following risks: adverse reactions from the intervention (such as pain, itching, mild transient skin redness and discomfort in the stimulated area, moderate fatigue, difficulty concentrating, and headaches), psychological discomfort during the assessment process, and distress from being asked sensitive questions. These are common risks in randomized clinical trials, but your information will be strictly confidential. Those who are unwilling to bear the risks of being assigned to the control group can refuse to participate. In the event of serious adverse effects, appropriate medical and nursing care will be provided until the symptoms subside. You will not be charged any fees.

10 **Benefits**

You will not be required to pay any costs associated with this trial; the study will provide you with feedback on your positive symptoms, negative symptoms, cognitive status, and other scale assessments and examinations, helping you to gain a better understanding of yourself. Additionally, participating in this study may potentially accelerate your recovery from psychiatric symptoms and cognitive functions, among other conditions.

11 **Withdrawal of consent after participation in study**

Your participation in this study is entirely voluntary. You may refuse to participate in the study, and declining to participate or withdrawing from the study at any time will not affect your current treatment, legal status, social services, or rights. There are two situations in which you might withdraw from the study early: one is if your doctor decides to terminate your participation for your benefit; the other is if you do not fully comply with the study protocol, in which case the researchers may also decide to discontinue your participation.

12 **If you have any questions about this research**

If you have any questions about this study, please contact our researchers:

Peng Luo	Email: 50230101138@stu.xxmu.edu.cn

Shanyuan He	Email: 50240101132@stu.xxmu.edu.cn

Hanshuo Su	Email: 50240101121@stu.xxmu.edu.cn

However, we may not be able to respond to you or answer your questions when they involve the privacy of other participants or intellectual property rights.

13 **The system of this study**

Name of the medical institution: Department of Psychiatry, The Second Affiliated Hospital of Xinxiang Medical University.

Principal Investigator names: Yan Ge Wei, MD; Peng Luo, Medical Graduate Student; Shanyuan He, Medical Graduate Student; Hanshuo Su, Medical Graduate Student.

**Signing of Informed Consent Form**

I have read this informed consent form.

I have had the opportunity to ask questions and all my questions have been answered.

I understand that participation in this study is voluntary.

I can choose not to participate in this study, or withdraw at any time by notifying the researcher without discrimination or retaliation, and my medical treatment and rights will not be affected.

If I need other treatments, or if I do not follow the research plan, or if there is any research-related injury or for any other reason, the research physician can terminate my continued participation in this study.

Finally, I decide to consent to participate in this study.

Subject's Signature: Date: ______ year ______ month ______ day

Researcher's Statement: I have accurately informed the subject of this document, he/she has read this informed consent form accurately, and I certify that the subject has had the opportunity to ask questions. I certify that he/she is voluntarily consenting.

Researcher's Signature: Date: ______ year ______ month ______ day
